# Parotid Gland Stem Cell Preservation during Intensity-Modulated Radiotherapy for Nasopharyngeal Carcinoma: Dosimetric Analysis and Feasibility

**DOI:** 10.1155/2022/4922409

**Published:** 2022-07-12

**Authors:** Lirong Zheng, Qiaoli Mei, Yuxiang Gao, Fenglei Du, Lin Xiao, Gangfeng Wu

**Affiliations:** ^1^Department of E.N.T., Taizhou Hospital of Zhejiang Province Affiliated to Wenzhou Medical University, Taizhou 317000, Zhejiang, China; ^2^Department of Pharmacy, Taizhou Hospital of Zhejiang Province Affiliated to Wenzhou Medical University, Taizhou 317000, Zhejiang, China; ^3^Department of Radiotherapy, Cancer Hospital of the University of Chinese Academy of Sciences (Zhejiang Cancer Hospital), Hangzhou 310000, Zhejiang, China; ^4^Department of Otolaryngology, Taizhou Enze Medical Center (Group) Enze Hospital, Taizhou 317000, Zhejiang, China; ^5^Department of E.N.T., Tai Zhou Second People's Hospital, Taizhou 317000, Zhejiang, China

## Abstract

**Objective:**

Parotid gland (PG) is a radiosensitive organ, and xerostomia (XS) is a key factor affecting patients' life quality after conventional radiotherapy for head and neck tumors. In this study, dosimetry analysis was performed on PG stem cell preservation in intensity-modulated radiotherapy (IMRT) for nasopharyngeal carcinoma (NPC).

**Methods:**

All clinical data of 80 NPC patients diagnosed pathologically in the Radiotherapy Department of Taizhou Hospital of Zhejiang Province Affiliated with Wenzhou Medical University from August 2017 to September 2019 were retrospectively analyzed. Patients were assigned to a regular group and a restricted group according to different IMRT plans, in which a dose limitation for the parotid duct was added in the restricted group in addition to the conventional plan used in the regular group to minimize the parotid duct radiation dose. The differences in planning target volume (PTV) dose distribution, organ at risk (OAR) dose, and dose to the PG and its ducts were compared between the two groups.

**Results:**

Significantly higher mean irradiation doses of the brainstem, mandible, and oral cavity were determined in the restricted group compared with the regular group (*P* > 0.05), but there was no significant difference in the mean dose of other OARs irradiated (*P* > 0.05). As compared to the irradiation of bilateral PGs, no statistical differences were found in the mean irradiation dose and V_30_ between regular and restricted groups (*P* > 0.05), but lower V_20_ and higher V_45_ were determined in the restricted group (*P* < 0.05). The mean irradiation dose, V_15_, V_20_, and V_26_ of bilateral parotid ducts were lower in the restricted group as compared to the regular group (*P* < 0.05).

**Conclusion:**

IMRT for NPC can effectively reduce the mean irradiation dose and play a PG stem cell preservation role by giving specific dose limitation conditions to the parotid duct area without affecting PTV dose distribution and OAR irradiation dose, which has certain feasibility.

## 1. Introduction

Nasopharyngeal carcinoma (NPC) is a malignancy derived from nasopharyngeal epithelial cells [1, 2]. Though rarely occurred worldwide, the age-standardized rate of NPC in some areas such as southern China, Southeast Asia, and North Africa is 4–25 cases per 100,000 people, as indicated by the GLOBOCAN data [3, 4]. Histologically, it can be classified as either keratinizing squamous cell carcinoma (KSCC), nonkeratinizing squamous cell carcinoma (NKSCC), or undifferentiated/poorly differentiated carcinoma [5], among which, KSCC has a low proportion and is common in Western countries [6, 7]. NPC, as a kind of highly radiosensitive and chemosensitive tumor [8], is routinely treated by radiotherapy because of its challenging anatomical location, when in the absence of metastasis [9]. At present, intensity-modulated radiotherapy (IMRT) and chemotherapy can provide a cure for almost all stage I and stage II NPC patients, contributing to an overall five-year survival of 98% and 92%, a local recurrence-free five-year survival rate of 98% and 94%, and a distant metastasis-free survival rate of 98% and 91%, respectively [10].

Currently, IMRT is the mainstream radiotherapy practice for patients with NPC [11, 12]. Studies have shown that the higher the radiation dose in the planning target volume (PTV), the better the local disease control rate [13]. However, applying radiation to cancerous tissues will inevitably irradiate adjacent normal counterparts, resulting in a series of side effects. In the first few weeks of radiotherapy, exposure of salivary glands (SGs) to radiotherapy often causes a loss of glandular function, resulting in insufficient salivary secretion that can lead to various side effects, among which speech disturbance, altered taste, dysphagia, and xerostomia (XS) are the most commonly reported [14, 15]. In some animal models, it has been found that the mechanism of radiation-induced SG injury is selective damage to the plasma membrane of secretory cells after radiation exposure, the subsequent DNA damage and acinar progenitor cell death, and finally acinar cell lysis [16–19].

The three major SG organs, namely, the parotid gland (PG), sublingual gland, and submandibular gland, are the main organs that secrete saliva, with PG secretion accounting for 80% of the total [20]. Thus, to prevent or reduce the occurrence of radioactive XS, the protection of PGs is of utmost importance. Patients receiving radiotherapy for head and neck cancer often experience reduced survival, as well as a large number of symptoms that can greatly reduce their quality of life [21, 22]. Although artificial lubricants and drugs that stimulate residual function can be used to ameliorate the consequences of hypoxia, most of their effects are transient. Such management techniques do not address the root cause of the problem: the lack of functional salivary acinar cells, which are caused by radiation-induced stem cell sterilization. The stimulation of cell proliferation after radiotherapy can improve salivary secretion only when some tissues are preserved or the dose of SG is kept below a certain level [23].

Research by van Luijk et al. [24] shows that the most radiosensitive part of SG contains a large number of excretory ducts, which may be the source of salivary gland adult stem cells (SGASCs), and these unevenly distributed SGASCs may determine the degree and effect of SG injury recovery after radiotherapy. The ductal system of SG is divided into intercalated ducts, secretory ducts, and excretory ducts [25]. Of them, intercalated ducts are directly connected with the acinar and then flow into the secretory tubes. While, intercalated and secretory tubes are both located in the lobule of the gland; the next level of the secretory tube is the excretory tube, which is thin to thick in diameter and passes through the connective tissue between the lobules and finally into the general duct, where secretions are discharged into the mouth and mixed to form saliva [26]. SGASCs are generally in a relatively static state. Once SGs are damaged, the proliferation and differentiation functions of SGASCs are active, which plays a very important role in repairing the structure and function of SGs in the later stage of injury [27]. Therefore, it is necessary to clarify the relationship between volume threshold and irradiation dose to facilitate the design of IMRT plans. While ensuring the dose to the gross tumor volume, a special dose limitation should be applied to the larger excretory ducts to minimize the volume and dose of PGs irradiated, so as to accelerate PG functional recovery after radiotherapy. Herein, the effects of PG stem cell preservation on PG function in IMRT were preliminarily analyzed by comparing the differences in the parotid ducts and the overall volume dosimetry of PGs in different radiotherapy plans.

## 2. Data and Methods

### 2.1. Research Subjects

All clinical data of 80 NPC patients pathologically diagnosed in the radiotherapy department of Taizhou Hospital of Zhejiang Province Affiliated to Wenzhou Medical University from August 2017 to September 2019 were collected and analyzed retrospectively. According to different IMRT plans, patients were assigned to a regular group with 35 cases and a restricted group with 45 cases. Inclusion criteria were as follows: confirmed diagnosis by histopathology; no distant cervical lymph node metastasis, lymph nodes ≤ 10 mm; and complete clinical data. Exclusion criteria were as follows: those who have PGs or submandibular glands removed; those with liver, kidney, heart, and lung dysfunctions; those with other serious systemic diseases; and those with incomplete clinical data. The medical ethics committee at our hospital approved the study protocol. The two cohorts of patients showed comparability in general data, as given in Table 1.

### 2.2. Treatment Methods

#### 2.2.1. Delineation of PTV and Organs at Risk (OARs)

Each patient was placed in a comfortable supine position, and a U-shaped thermoplastic mask for the head was made, which was used together with the head frame to fix the patient's body position for CT scanning. After the scan, the CT images were transmitted to the treatment planning system of the workstation through the network to sketch the vital organs, followed by reconstruction, planning, calculation, and evaluation. The clinical PTV was delineated by referring to ICRU Reports 50 and 62 [28]. The nasopharyngeal GTVnx and cervical GTVnd were delineated according to the boundaries of the primary tumor and neck metastatic lymph nodes shown by CT and MRI. CTV1 was obtained by GTVnx extension of 5–10 mm and must include the entire mucosa of the nasopharyngeal cavity and 0.5 cm below it. CTV2 was delineated by expanding CTV1 by 5–10 mm, covering GTVnd and the lymphatic drainage area where it was located and which needed to be prevented from irradiation. The specific expansion distance of CTV1 and CTV2 was determined according to the actual situation and the surrounding tissue structure. When approaching the brainstem, spinal cord, and other vitals, the distance should be correspondingly reduced to 2-3 mm outside GTVnx and CTV1. The PTV of each target region was uniformly extended by 3.0 mm in all directions, and if it exceeded the skin, it was retracted to 2 mm below it.

#### 2.2.2. OARs and Parotid Ducts

According to Sun et al., the OARs were delineated, including the brainstem, spinal cord, lens, optic nerve, eyeball, optic chiasm, temporomandibular joint, temporal lobe, mandible, and PG.

Bilateral parotid main ducts and branch ducts were located by parotid duct angiography and CT. According to the high-density parotid duct displayed on CT images, the primary and first-class branch ducts in parotid tissue were delineated along their boundaries and named as ducts. If the medial boundary of the duct was less than 1 cm away from CTV2, it was modified properly to ensure a distance greater than 1 cm. PTV-ducts, which were uniformly expanded by 2.0 mm in the anterior, posterior, left, right, upper, and lower directions of the duct, were defined as OARs, to which dose limitation conditions were given when formulating the IMRT plan.

#### 2.2.3. Radiotherapy Plans

In the regular group, the radiation dose was set as follows: PTVnx: 70–72 Gy/30 times, PTVnd: 62–68 Gy/30 times, PTV1: 60–64 Gy/30 times, and PTV2: 54–56 Gy/30 times. The fraction doses were 2.32 Gy each time for PTVnx, 2.06–2.26 Gy each time for PTVnd, 2.0–2.12 Gy each time for PTV1, and 1.8 Gy each time for PTC2, all for five times a week. 95% of the above PTV was required to meet the prescription dose, the PTV receiving >110% of the prescribed dose should be <20%, the PTV receiving <93% of the prescribed dose should be <3%, and a dose >110% of the prescribed dose cannot appear anywhere outside PTV. OAR tolerance doses are given in Table 2.

Restricted group: based on the regular group, the doses of PTV-ducts were kept as low as possible on the premise of meeting the dose requirements of PTV and the dose limit of OARs, and the dose constraint for PGs was relaxed when necessary.

### 2.3. Evaluation Indicators

The dosimetric data of each target volume and OAR were collected to compare the dose distribution between the two plans. The following indicators were the primary endpoints of target volume: mean dose (*D*_mean_), the percentage volume receiving 95% (V_95_), 100% (V_100_), and 105% (V_105_) of the prescription dose. OARs were evaluated at mean doses.

### 2.4. Statistical Analysis

SPSS 22.0 statistical software package (IBM, Armonk, New York) was used to analyze the data. The enumeration data (denoted by *n* (%)), were analyzed by the *χ*^2^ test. The quantitative data that accorded with normal distribution were expressed as mean ± SD, and intergroup comparisons were done by the independent sample *t*-test and paired *t*-test, respectively. A one-way ANOVA was applied to compare the differences in each group over different doses of percentage volume followed by Turkey's post hoc test. The significance threshold was *P* < 0.05.

## 3. Results

### 3.1. Irradiation Dose of PTV in the Two Groups

In terms of target volume irradiation dose, the *D*_mean_ as well as V_95_, V_100_, and V_105_ of PTVnx, PTV1, and PTV2 were found to differ insignificantly between the regular group and the restricted group (*P* > 0.05), as given in Table 3.

### 3.2. PG and Its Duct Irradiation Doses in Two IMRT Plans

The *D*_mean_ of the left and right PGs was not statistically different between the regular group and the restricted group (*P* > 0.05). However, the restricted group showed lower V_20_ and higher V_45_ than the regular group (*P* < 0.05), but with no statistical difference in V_30_ (*P* > 0.05). After the bilateral parotid ducts were subjected to specific dose restriction in the restricted group, the *D*_mean_, V_15_, V_20_, and V_26_ were significantly lower than those in the regular group (*P* < 0.05), as given in Table 4.

### 3.3. Mean Radiation Dose to Other OARs in IMRT Plans of the Two Groups

As to the mean radiation doses to OARs, the *D*_mean_ of the brainstem, oral cavity, and bilateral mandibles was statistically different between groups (*P* < 0.05). However, a significant difference was absent in the mean dose of exposure to other OARs such as the spinal cord, temporal lobe, temporomandibular joint, lens, optic nerve, eyeball, middle ear, submandibular gland, and throat (*P* > 0.05), as given in Table 5.

## 4. Discussion

Among SGs, PGs produce 60–65% of the total saliva [29]. Due to the high dose of bilateral PG irradiation caused by conventional two-field radiotherapy during the treatment of NPC, PGs are inevitably damaged, which leads to a significant decline in PG secretion function, as well as acute and chronic sequelae in patients such as XS that is difficult to recover by itself with no effective treatment at present other than prevention [30]. Even when image-guided techniques are used, XS can be caused by an increase in the actual dose of PGs due to changes in organ anatomy, tumor size, and mass during radiotherapy. These changes will not only lead to insufficient target doses but also to additional complications with excessive doses applied to OARs [31, 32]. In this study, specific dose limits were applied to the parotid duct area to protect parotid stem cells. The larger excretory duct area of PGs is small in size relative to the total volume of PGs, making it easier to give a strict dose limit without affecting the dose distribution of the entire plan, which is a feasible method.

In this study, two sets of IMRT plans were adopted. The main difference is that the regular group did not restrict parotid duct irradiation dose, but the restricted group did, so that the dose of PG ducts could be reduced as much as possible without affecting the PTV dose distribution or the dose to OARs. The results showed that there were no significant differences in *D*_mean_, V_95_, V_100_, and V_105_ of PTVnx, PTV1, and PTV2 of PTV. Chao [33] believed that IMRT can significantly reduce the irradiation dose of bilateral PGs while not reducing the therapeutic effect, thus significantly reducing the late SG injury. The mean dose of brainstem, mandible, and oral cavity was found to be statistically higher in the restricted group compared with the regular group, but no distinct difference was observed in the average dose of other OARs. It may be because parotid ducts are located on the left and right sides, and when they are given a strict dose limit, the radiation intensity in the anterior and posterior directions of the patient will increase, resulting in an increase in *D*_mean_ of the tissue at the same level as the ducts, such as the brainstem, mandible, and oral cavity. But the increase of relevant indexes in this study is not significant and does not exceed the limit requirements of OARs. In addition, the mean dose and V_30_ of bilateral PGs showed no statistical differences between the two groups, but lower V_20_ and higher V_45_ were determined in the restricted group. Since the parotid duct is located in the center of the parotid tissue, the decline of parotid duct dose is bound to be accompanied by the decrease of the dose of parotid tissue near the duct, which can explain the lower V_20_ in the restricted group compared with the regular group. In the process of optimization, the target volume coverage of PTV2 needs to be compensated, so that the PG dose will be correspondingly increased at the level of parotid tissue far away from the duct, thus avoiding the serious underdose of PTV2. Therefore, V_45_ increased and V_20_ decreased in PGs in the restricted group, while *D*_mean_ and V_30_ showed no significant difference. Münter et al. [34] used IMRT to treat 18 patients with head and neck cancer. The results indicate that the dose for PG function preservation should be no higher than 26–30 Gy, and when taking different methods, time, radiotherapy techniques, and statistical models of saliva measurement into consideration, the optimal radiation dose of PGs should be 25–35 Gy.

In this study, the mean dose of PG exposure in both groups was within the standard range of optimal radiation dose. We also found significantly lower *D*_mean_, V_15_, V_20_, and V_26_ of bilateral parotid ducts in the restricted group as compared to the regular group. The above results suggest that specific dose limitation of the parotid duct in IMRT can effectively reduce its exposure dose. However, the volume of the parotid duct will shrink during radiotherapy and its position will change greatly due to weight loss and other reasons. Some research results showed that the PG volume decreased at the end of fractionated radiotherapy, with an average volume reduction of 21.3–42% and an average reduction rate of 0.4–1.4%/day [35, 36]. Vásquez Osorio et al. [37] analyzed 10 patients with head and neck tumors and also concluded that the SG volume of patients decreased significantly at the end of radiotherapy, including 17 ± 7% reduction of PGs and 20 ± 10% reduction of submandibular glands. However, this study still has room for improvement. As the PG shrinks during radiotherapy, the position of the duct changes. In this study, the PTV-duct was evenly expanded by 2 mm in all directions based on the parotid duct, aiming to reduce the influence of the position change of the parotid duct. Nevertheless, it is difficult to ensure that the dose-limiting area set in the radiotherapy plan can accurately land on the parotid duct. Therefore, in future studies, attempts can be made to find better ways to optimize repositioning and target area redelineation during radiotherapy, to minimize the influence of factors affecting the position of the parotid duct and optimize radiotherapy protocols.

To sum up, by giving parotid ducts specific dose limits, the dose of a few OARs will be slightly increased within the allowable range, and the dose of parotid ducts will be significantly reduced, which better protects parotid stem cells of the parotid ducts, creates conditions for structural and functional recovery of parotid tissue after radiotherapy, and lays a foundation for better prevention of radiation-induced XS.

## Figures and Tables

**Table 1:** Comparison of general information.

	Regular group (*n* = 35)	Restricted group (*n* = 45)	*χ* ^2^/*t*	*P*
Sex			0.9786	0.3225
Male	24 (68.6)	26 (57.8)		
Female	11 (31.4)	19 (42.2)		

Age	39.7 ± 3.3	40.3 ± 3.0	0.8494	0.3983

Clinical staging			0.3304	0.9542
T1	8 (22.9)	11 (24.4)		
T2	12 (34.3)	14 (31.1)		
T3	8 (22.9)	9 (20.0)		
T4	7 (20.0)	11 (24.4)		

Histological classification			0.1055	0.7453
Undifferentiated nonkeratinizing carcinoma	29 (82.9)	36 (80.0)		
Differentiated nonkeratinizing carcinoma	6 (17.1)	9 (20.0)		

**Table 2:** Tolerated doses for organs at risk.

Organs at risk	Dose limits
Brainstem	*D*_max_ < 54 Gy, 1% vol < 60 Gy
Spinal cord	*D*_max_ < 45 Gy, 1% vol < 50 Gy
Optic nerve	*D*_max_ < 54 Gy
Eyeball	*D*_max_ < 54 Gy
Lens	*D*_max_ < 10 Gy
Temporal lobe	*D*_max_ < 72 Gy
Temporomandibular joint	*D*_max_ < 70 Gy
Parotid gland	*D*_mean_ < 26 Gy or 50% vol < 30 Gy
Submaxillary gland	>20 cm^3^ vol received <20 Gy
Mandible	*D*_max_ < 70 Gy
Middle ear	*D*_mean_ < 50 Gy
Throat	*D*_mean_ < 45 Gy
Oral cavity	*D*_mean_ < 40 Gy

*D*_max_ = maximum dose; *D*_mean_ = mean dose.

**Table 3:** Comparison of irradiation dose in planning target volume between two intensity-modulated radiotherapy plans.

Target volume	Regular group (*n* = 35)	Restricted group (*n* = 45)	*t*	*P*
PTVnx				
*D*_mean_ (Gy)	72.03 ± 2.22	72.26 ± 2.86	0.3924	0.6958
V_95_ (%)	99.67 ± 0.16	99.66 ± 0.09	0.3536	0.7246
V_100_ (%)	95.41 ± 1.32^*∗*^	94.98 ± 1.23^*∗*^	1.5022	0.1371
V_105_ (%)	15.40 ± 4.25^*∗*#^	15.78 ± 4.34^*∗*#^	0.3920	0.6961

PTV1				
*D*_mean_ (Gy)	68.78 ± 4.90	68.18 ± 5.20	0.5249	0.6011
V_95_ (%)	97.51 ± 1.05	97.05 ± 1.35	1.6617	0.1006
V_100_ (%)	94.04 ± 3.69^*∗*^	93.91 ± 3.49^*∗*^	0.1612	0.8724
V_105_ (%)	65.85 ± 7.37^*∗*#^	66.47 ± 9.15^*∗*#^	0.3267	0.7448

PTV2				
*D*_mean_ (Gy)	60.21 ± 2.01	59.96 ± 2.12	0.4648	0.6434
V_95_ (%)	96.78 ± 1.18	96.25 ± 1.03	1.4659	0.1467
V_100_ (%)	92.21 ± 3.05^*∗*^	91.97 ± 2.99^*∗*^	0.6202	0.5369
V_105_ (%)	52.87 ± 5.22^*∗*#^	53.06 ± 5.54^*∗*#^	0.2264	0.8214

^*∗*^ *P* < 0.05 vs. V_95_; ^#^*P* < 0.05 vs. V_100_.

**Table 4:** Comparison of the parotid gland and its duct radiation doses between two intensity-modulated radiotherapy plans.

Organs at risk	Regular group (*n* = 35)	Restricted group (*n* = 45)	*t*	*P*
Left parotid gland				
*D*_mean_ (Gy)	31.55 ± 0.77	31.32 ± 0.92	1.1896	0.2378
V_20_ (%)	78.77 ± 3.22	66.12 ± 3.81	15.7451	**<0.0001**
V_30_ (%)	43.26 ± 3.13	43.27 ± 3.55	0.0132	0.9895
V_45_ (%)	24.64 ± 2.72	26.87 ± 2.34	3.9378	**0.0001**

Left ducts				
*D*_mean_ (Gy)	22.28 ± 1.33	15.38 ± 0.60	31.0195	**<0.0001**
V_15_ (%)	98.61 ± 0.30	39.11 ± 9.91	35.4573	**<0.0001**
V_20_ (%)	72.22 ± 9.47	2.47 ± 0.39	49.4449	**<0.0001**
V_26_ (%)	14.80 ± 5.46	0.14 ± 0.05	18.0435	**<0.0001**

Right parotid gland				
*D*_mean_ (Gy)	31.03 ± 0.93	30.91 ± 0.97	0.5588	0.5778
V_20_ (%)	75.82 ± 4.64	61.75 ± 4.00	14.5498	**<0.0001**
V_30_ (%)	42.45 ± 3.73	42.61 ± 3.46	0.1983	0.8433
V_45_ (%)	22.69 ± 3.09	26.70 ± 3.11	5.7372	**<0.0001**

Right ducts				
*D*_mean_ (Gy)	22.26 ± 1.27	14.86 ± 0.41	34.3638	**<0.0001**
V_15_ (%)	98.88 ± 0.74	37.47 ± 14.62	24.7901	**<0.0001**
V_20_ (%)	73.84 ± 16.69	1.85 ± 0.22	28.9848	**<0.0001**
V_26_ (%)	12.02 ± 5.16	0.10 ± 0.02	15.5248	**<0.0001**

The bold text means statistically significant.

**Table 5:** Comparison of mean irradiation doses of other organs at risk in IMRT plans of the two groups.

Organs at risk	Regular group (*n* = 35)	Restricted group (*n* = 45)	*t*	*P*
Spinal cord	21.95 ± 3.54	21.74 ± 4.37	0.2313	0.8177
Brainstem	29.25 ± 2.19	30.31 ± 2.10	2.1981	**0.0309**
Left temporal lobe	18.79 ± 3.12	18.55 ± 3.41	0.3240	0.7468
Right temporal lobe	19.06 ± 2.39	19.58 ± 3.39	0.7703	0.4435
Left temporomandibular joint	38.54 ± 3.40	39.05 ± 3.65	0.6386	0.5249
Right temporomandibular joint	39.61 ± 3.28	39.47 ± 3.32	0.2136	0.8314
Left lens	5.28 ± 1.26	5.31 ± 1.24	0.3531	0.7249
Right lens	5.47 ± 1.68	5.53 ± 1.71	0.1348	0.8931
Left optic nerve	33.07 ± 2.77	32.85 ± 2.45	0.3763	0.7077
Right optic nerve	34.55 ± 3.05	34.71 ± 3.34	0.2207	0.8259
Left eyeball	11.17 ± 1.67	11.26 ± 2.08	0.2088	0.8351
Right eyeball	10.83 ± 1.70	10.92 ± 1.35	0.3520	0.7258
Left submandibular gland	38.24 ± 4.26	38.64 ± 4.38	0.7219	0.4725
Right submandibular gland	38.11 ± 3.97	38.18 ± 3.94	0.0553	0.9560
Left middle ear	44.87 ± 6.41	44.62 ± 7.05	0.1441	0.8857
Right middle ear	45.68 ± 5.47	45.41 ± 5.53	0.2095	0.8346
Left mandible	34.08 ± 2.32	35.22 ± 2.09	2.3063	**0.0237**
Right mandible	34.91 ± 1.96	36.06 ± 2.66	2.1437	**0.0352**
Throat	32.30 ± 2.79	32.49 ± 3.39	0.2683	0.7892
Oral cavity	31.36 ± 2.96	33.63 ± 3.64	2.9972	0.0036

The bold text means statistically significant.

## Data Availability

The dataset used to support the findings of this study is available from the corresponding author upon request.
